# Impella-Supported Surgical Repair for Acute Phase Posterior Ventricular Septal Rupture

**DOI:** 10.1016/j.atssr.2023.11.023

**Published:** 2023-12-12

**Authors:** Motoki Nagatsuka, Tohru Asai, Kenichiro Noguchi, Tsuyoshi Yamabe, Yusuke Gunji, Daisuke Hama

**Affiliations:** 1 Department of Cardiovascular Surgery, Shonan Kamakura General Hospital, Kamakura-shi, Kanagawa, Japan

## Abstract

A 72-year-old man was diagnosed with posterior ventricular septal rupture after acute myocardial infarction with dissection in the posterior wall of the ventricular septum. On admission, cardiogenic shock was identified, and an Impella CP was inserted to improve his hemodynamics. Extended sandwich patch repair through the right ventricle with a Dacron patch was successfully performed, achieving an excellent postoperative course with stable hemodynamics. Preoperative Impella introduction may be a useful strategy for ventricular septal rupture repair in the acute phase.

Ventricular septal rupture (VSR) is one of the most serious complications of acute myocardial infarction. The risk of surgical mortality is high in the acute setting and is complicated by cardiogenic shock, as shown in a study on The Society of Thoracic Surgeons database, in which there was a 54.1% mortality rate for repair attempts within 7 days after myocardial infarction.^1^ This shows the lack of improvement from the 52% surgical mortality rate within 3 weeks of onset reported by Daggett and coworkers in 1977.^2^ These patients tend to be hemodynamically unstable, intubated preoperatively, and suffering from multiorgan failure. In addition, the repair technique itself is technically difficult because of the fragile necrotic myocardium. In these patients, mechanical circulatory support devices, such as venoarterial extracorporeal membrane oxygenation and Impella (Abiomed), may be useful to stabilize hemodynamics, to improve insufficient peripheral circulation, and to extend the surgical wait time.^3^ However, clear criteria for which patients should undergo emergency VSR repair and which should receive mechanically assisted bridging therapy remain lacking. We describe herein the case of a patient who underwent VSR repair in the acute phase under Impella support and achieved a good postoperative course.

A 72-year-old man was brought to our hospital with chest pain and syncope. Transthoracic echocardiography showed left-to-right shunt with dissection in the posterior wall of the ventricular septum (Figure 1), and electrocardiography showed ST-segment elevation in the II, III, and aVF leads. The patient was diagnosed with posterior VSR associated with acute myocardial infarction. Coronary angiography revealed complete occlusion of the proximal right coronary artery in addition to a 90% stenosis in the mid left anterior descending artery and another 90% stenosis in a large obtuse marginal artery. The serum concentration of lactate was 16.28 mmol/L on admission. The patient further decompensated into cardiogenic shock, so an Impella CP device was inserted. The Impella CP was driven by P2 because of concerns about right-to-left shunt.

**Figure 1:**
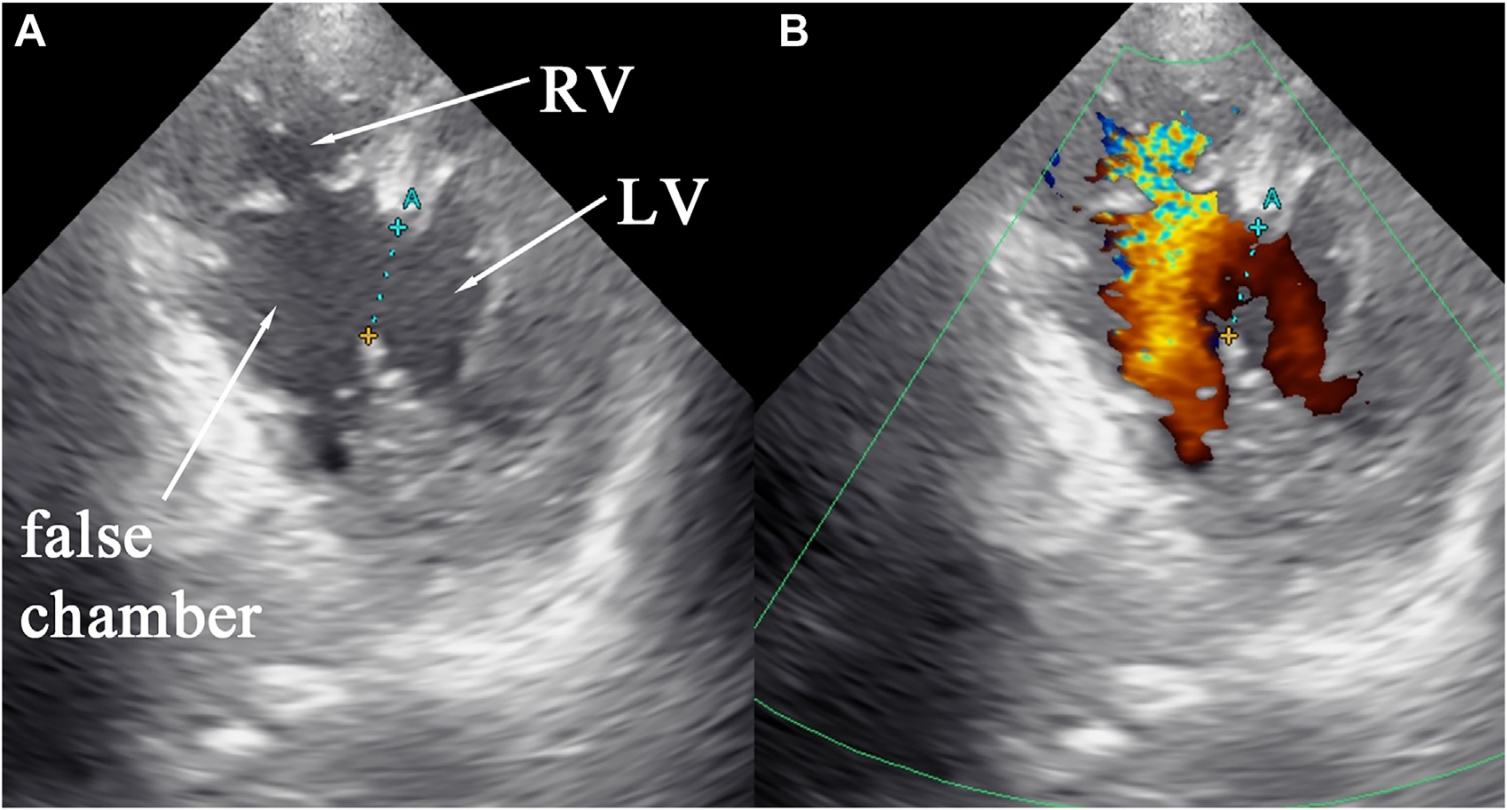
Short-axis transthoracic echocardiography. (A) Echocardiography shows the right ventricle (RV), the left ventricle (LV), the false chamber, and an 18-mm defect in the posterior ventricular septum. (B) Ventricular septal rupture continuous with the large dissected space is also continuous with the true lumen of the right ventricle.

After induction of general anesthesia, a median sternotomy was performed. Extracorporeal circulation was established by withdrawing blood from the bi-vena cava and returning the blood through the ascending aorta. After cross-clamping of the ascending aorta, cold-blood cardioplegia was induced with the injection of cardioplegic solution in antegrade and retrograde manners into the coronary arteries. A trans–right ventricular approach was used, incising the right side of the posterior descending branch. The head of the Impella CP could not be identified, and the false chamber with dissected ventricular septum was seen. Incision through the septum revealed the head of the Impella CP. Extended sandwich patch repair^4^ was performed with 3-0 polypropylene 8-stitch interrupted mattress sutures and a 5 × 6-cm Dacron patch (Figure 2). Coronary artery bypass grafting (saphenous vein graft to the left descending artery, saphenous vein graft to the obtuse marginal artery) was performed under cross-clamping of the aorta. Withdrawal from cardiopulmonary bypass was smooth, and the patient was switched from the Impella to an intra-aortic balloon pump.

**Figure 2:**
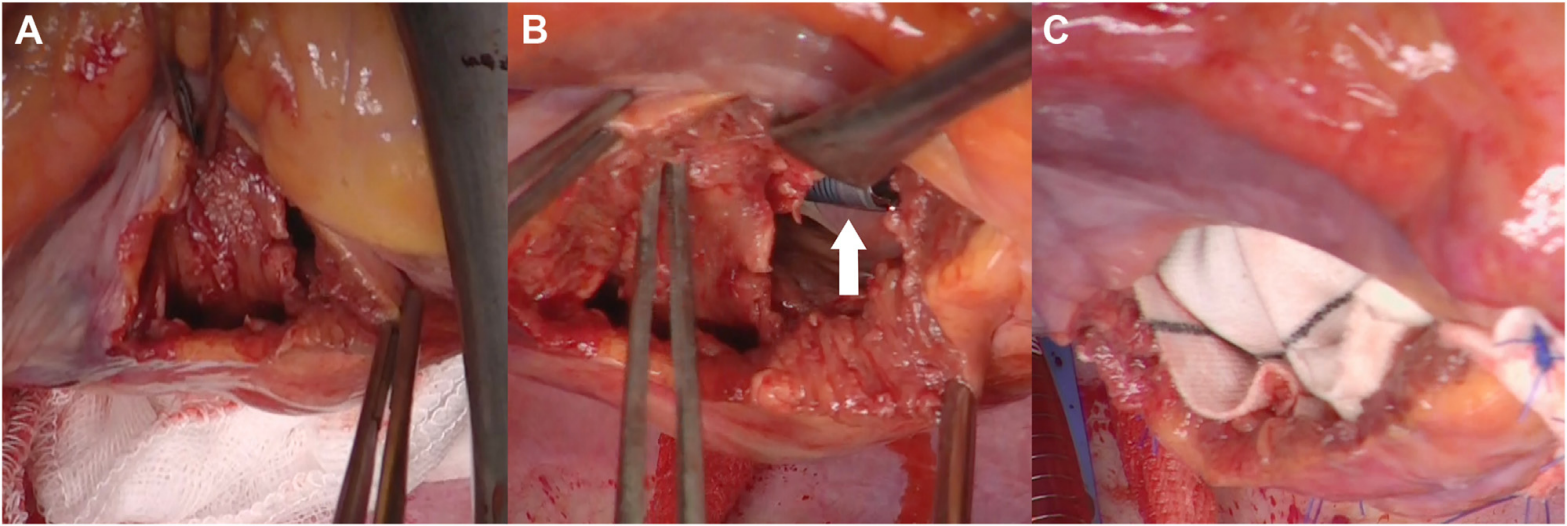
Intraoperative images. (A) Dissected ventricular septum in the false chamber as seen by a right ventricular approach. (B) After the dissected ventricular septum was incised and the Impella device (arrow) was positioned in the left ventricular cavity, (C) the ventricular septal rupture was repaired by an extended sandwich patch technique with a Dacron patch.

The patient was returned to the intensive care unit with stable hemodynamics. He was transferred to a rehabilitation hospital on postoperative day 22.

## Comment

In the acute phase of myocardial infarction, the myocardium is fragile and the boundary between normal and infarcted myocardium is unclear. This may result in insufficient exclusion and a residual shunt. Good surgical outcomes have therefore been reported by waiting for the infarcted myocardium to stabilize before surgery, but surgical intervention is unavoidable in some cases when the shunt volume is too large and complicated by cardiogenic shock. The key is therefore to achieve stable surgical results even in cases in which hemodynamic status cannot be maintained. However, the improved outcomes with delayed surgery may be related to the evolution of the infarct and improved stability of the cardiac tissue, allowing more effective repair, but may also reflect survival bias because early surgery is usually performed for individuals with marked hemodynamic instability and circulatory compromise.

**Figure 3:**
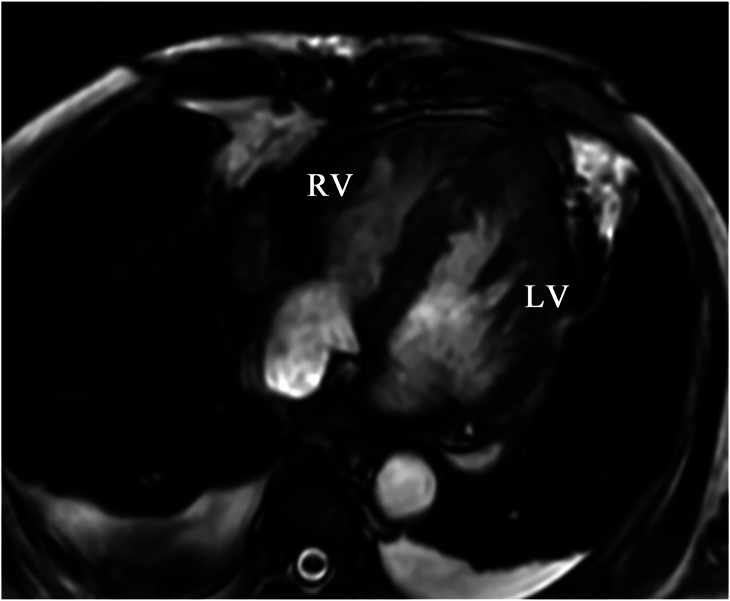
Nonenhanced magnetic resonance imaging of the heart shows no residual interventricular shunt. (LV, left ventricle; RV, right ventricle.)

In this study, we performed VSR repair through a right ventricular approach under Impella support in a patient who was unable to maintain stable hemodynamics. This patient regained hemodynamic stability immediately after the procedure and showed no residual shunt (Figure 3). Kinoshita and colleagues^5^ reported an in-hospital mortality rate of 100% (6/6) after emergency surgery for VSR with serum lactate concentration >10 mmol/L due to preoperative circulatory failure, indicating that preoperative lactate level may represent a prognostic factor. Serum lactate concentration in this case was 16.28 mmol/L on admission but had decreased to 8.31 mmol/L by the time general anesthesia was induced, approximately 3 hours after starting of Impella support. The patient had myocardial infarction with a right coronary artery as the culprit vessel, complicated by right-sided heart failure, and the occurrence of right-to-left shunt was a concern. The advantage of the Impella is the left ventricular venting effect, but this may induce right-to-left shunt in patients with right-sided heart failure, which may in turn require the addition of venoarterial extracorporeal membrane oxygenation. The strategy in this case was appropriate, given the aim of achieving a venting effect in the acute phase.

After VSR repair, if no residual shunt is present and weaning from cardiopulmonary bypass is not a problem, Impella support may not be necessary. The most frequent complications associated with Impella use are major bleeding at the vascular access site, hemolysis, and limb ischemia. A common complication with prolonged Impella use is hemolysis, which has been reported within the first 24 hours of use in 5% to 10% of patients.^6^ Persistent hemolysis associated with acute kidney injury is an indication for device removal. The risk of these complications is minimized if Impella support is provided only in the acute preoperative phase. As a secondary factor, the perforated septum is difficult to recognize in cases of complicated anatomy with myocardial dissection, but checking the Impella facilitates an understanding of the anatomy. VSR with myocardial dissection is reportedly often seen in the posterior wall,^7^ making the anatomy difficult to identify. This may complicate the repair and prolong the duration of cardiopulmonary bypass. In this case, the patient had a complicated posterior VSR with myocardial dissection and severe circulatory failure, but the Impella stabilized the preoperative hemodynamics by venting the left ventricle, and a good outcome was obtained. Preoperative Impella implantation may be a useful strategy for VSR repair in the acute phase.
